# Metal-on-metal hip implants: Revisiting degradation product chemistry and molecular mechanisms of ARMD

**DOI:** 10.1016/j.bbiosy.2026.100134

**Published:** 2026-03-18

**Authors:** Rolando A. Gittens, Venettia R. Leslie, David J. Cohen, Zvi Schwartz, Barbara D. Boyan

**Affiliations:** a Center for Biodiversity and Drug Discovery, Instituto de Investigaciones Científicas y Servicios de Alta Tecnología (INDICASAT AIP), Panama, Republic of Panama; b Centro de Investigación e Innovación Educativa, Ciencia y Tecnología (CiiECYT-AIP), Panama, Republic of Panama; c Research & Innovation Division, Instituto Técnico Superior Especializado (ITSE), Panama, Republic of Panama; d School of Medicine, Universidad de Panama, Panama, Republic of Panama; e Imaging and Radiology Department, Complejo Hospitalario Dr. Arnulfo Arias Madrid-CSS, Panama, Republic of Panama; f Department of Biomedical Engineering, Virginia Commonwealth University, Richmond, Virginia, USA

**Keywords:** Nanoparticles, Wear debris, Macrophage, Clearance, Hip implant failure, In vitro model, Degradation products, Metal corrosion

## Abstract

Over the past decade, publications, legal cases, and regulatory panels have intensified scrutiny of implant degradation products—particularly nanoparticles and chemically reacted corrosion products—in the failure of total hip prostheses. While orthopedics has long linked wear to loosening, the mechanisms and exposure conditions that drive adverse local tissue reactions remain incompletely defined. Historically, microscale polymer particles were considered the primary drivers of inflammation; however, reductions in polyethylene wear have made nanoscale species and metal degradation products more prominent around some implants. Advances in analytical techniques now show that “metal debris” often consists of oxidized chromium(III) oxides/phosphates with cobalt largely present as soluble or complexed species, and that modular taper junctions can contribute mixed degradation products that differ from bearing-surface debris. Clinically, these issues remain relevant because many patients implanted during the peak adoption period of metal-on-metal (MoM) systems still live with these devices and may present across diverse health systems, where prior implant records and standardized surveillance may be incomplete. Unfortunately, in vitro studies have not always been as informative as expected because static, bolus exposures and idealized particles fail to capture in vivo speciation, dose rate (flux), protein corona formation, and physiologic transport. This review summarizes current knowledge on particle size distributions and degradation product chemistry in hip arthroplasty and discusses how these variables shape molecular and cellular pathways implicated in ARMD, while outlining experimental strategies that better reproduce physiological exposure conditions.

## Highlights

• ARMD risk depends on source, speciation, and dose/flux—not “metal debris” alone.
• Bearing and taper junctions may yield distinct chemical fingerprints; key gap.
• Realistic in-vitro studies require speciation-matched, flux-controlled exposures under flow.
• Single-cell/spatial omics could map pathways (NLRP3, HIF-1α) to tissue niches.

## Graphical abstract

**Figure:**
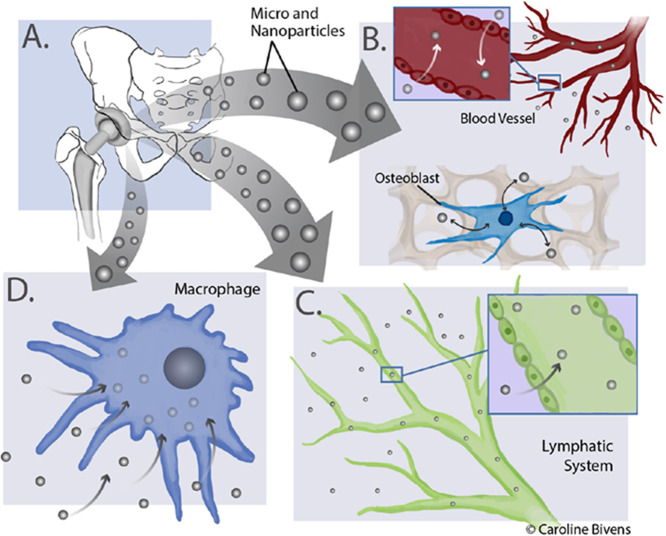

## 1 Introduction

Hip arthroplasty, also known as hip replacement, is a medical procedure that involves surgically removing a diseased or injured hip joint and replacing it with a prosthetic implant. Most patients require hip replacement as a consequence of osteoarthritis, a debilitating disease in which the thin layers of cartilage that cover the outermost surface of the femur in the hip socket become degraded, causing severe pain and mobility impairment [1,2]. The goal of most hip replacements is to restore joint function and range of motion and to relieve pain. Ideally, this could be done by fixing or regenerating the cartilage layer that naturally provides such properties, and the most recent efforts in new fields such as tissue engineering and cellular therapy are trying to do just that [3,4]. However, these therapies are still years away from being routinely offered in clinics with consistently effective results [5].

Current implant technology has been termed the “operation of the century” [6,7], providing an excellent alternative for restoring joint function and quality of life for patients. Still, with the technologies of today no artificial joint is as good as the natural joint, as evidenced by the expected percentage of unsuccessful implantations and the instances of recalls for hip implant models due to possible design failures [[8], [9], [10], [11]]. It is well recognized in the orthopedic community that all total hip replacements experience wear and generate particles. However, survival rates above 95% at 10 years and over 77% at 20 years [[12], [13], [14], [15], [16]], with sustained improvements in the last few years [17], makes evident that not all wear debris and degradation/corrosion products renders the implant unsuccessful. We do not yet have an in-depth understanding of the underlying mechanisms that determine the biological response to the degradation products to help elucidate the role of metal ions, nanoparticles or other phenomena in failure. Still, several clinical and biological factors have been well documented and associated with aseptic implant failures such as excessive innate and adaptive immune reactivity to implant degradation products [[18], [19], [20], [21]]; aseptic, lymphocyte-dominated vasculitis-associated lesions (ALVAL) [22,23]; pseudotumor / metal toxicity [24,25]; metal hypersensitivity [[26], [27], [28]], and a combination of all the above generally referred to as adverse local tissue reactions to metal debris (ARMD or ALTR) [[29], [30], [31]].

Although primary implantation of MoM bearings has declined following regulatory actions, MoM-related degradation remains clinically relevant because many patients implanted during the 2003–2010 adoption peak still retain these devices, and similar corrosion-driven mechanisms are observed at modular junctions in non-MoM constructs. As these devices exceed 15 years in situ, they are prone to distinct late-stage failure modes, particularly "trunnionosis"—a specific form of mechanically assisted crevice corrosion at the head-neck taper [32].

Complicating this clinical landscape is the increasing mobility of the aging population. Through the phenomenon of International Retirement Migration (IRM), large cohorts of retirees are moving from nations where MoM was prevalent to emerging medical hubs in Latin America and Southeast Asia [33,34]. Panama serves as a prime example of this trend, hosting a growing population of North American and European retirees who frequently report fragmented care and a lack of medical record transfer [35]. For local orthopedic surgeons who may not have participated in the initial MoM implantation wave, distinguishing the soft-tissue destruction of Adverse Reactions to Metal Debris (ARMD) from septic loosening is a diagnostic minefield. Without a clear understanding of the unique biological footprint of metal degradation products, specifically the ion thresholds associated with ARMD risk [36,37], these patients risk being treated misguidedly (e.g., infection) or simply late, while the underlying tribocorrosion continues unchecked.

Our study presents a narrative, mechanism-focused review. We searched PubMed/Medline and Scopus for English-language articles (2000–2026) using combinations of keywords including “metal-on-metal“, “tribocorrosion“, “fretting corrosion“, “trunnionosis“, “cobalt“, “chromium phosphate“, “wear nanoparticles“, “ALTR/ARMD“, and “ALVAL“. We prioritized retrieval/tissue-chemistry studies, tribology/tribocorrosion investigations, and mechanistic immunology papers reporting particle size distributions, speciation, dose metrics, or clinically linked tissue outcomes. Reference lists of key reviews were also screened to identify additional relevant primary studies. Unlike prior reviews that primarily summarize early clinical experience or general wear-biology, we integrate recent speciation-resolved tissue chemistry with pathway-level mechanisms and explicitly frame bearing versus taper sources as a testable comparative question.

## 2 Bearing surfaces and degradation products: historical background

### 2.1 First generation of total hip replacement implants

**Fig. 1:**
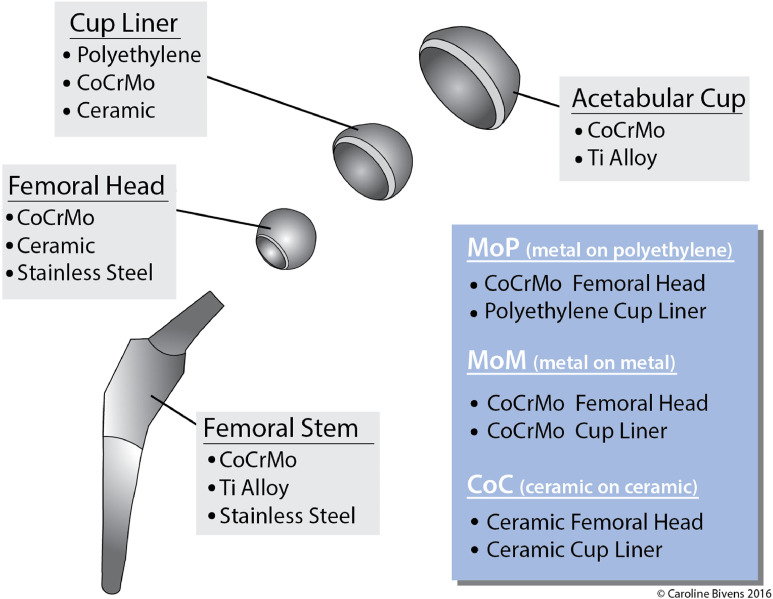
**Bearing surfaces of a hip replacement implant.** Schematic representation of the different components in a hip replacement implant.

Bearing surfaces have varied throughout the years (Fig. 1, Table 1), mainly driven by the experience of the orthopedic surgeons and the long-term outcomes of the patients. Still in use today, initial hip arthroplasty efforts attempted to replace only the femoral head or the acetabulum, in effect creating a bone/implant bearing surface, but had high revision rates during the initial two years in the patient [38,39]. The first generation of metal-on-metal (MoM) implants was introduced around 1960 to achieve low frictional resistance, reduce pain and improve range of movement in the hip joint [40,41]. The outcomes of MoM implants were considered outstanding at that time, with 94% of patients assessing their results “excellent” or “good”, when the definition of “good” meant free from pain, 50% range of movement, a slight limp and the use of “one stick” (i.e., cane) [40,42]. Metal sensitivity was reported in a small percentage of patients, attributed to metal ions released from the implant [[43], [44], [45]].

At about the same time, the first generation of metal-on-polyethylene (MoP) implants was also being evaluated, since previous trials with polytetrafluoroethylene (PTFE) exhibited considerably high wear volumes (linear wear rate of ∼2.26 mm / year) and elicited tissue reactions [46]. The good results achieved with MoP implants caused them to be favored over MoM devices by the mid-1970s and until the late 1980s, although long term survival rates were similar [47,48].

The low-friction design of MoP implants (linear wear rates of ∼0.05 mm / year [49] to 0.103 mm / year [50], Table 1) required the use of a small femoral metal head (22.25 mm) with an acetabulum cup twice in radius and lined with ultra-high molecular weight polyethylene (UHMWPE) [51,52]. This combination led to a high incidence of dislocations and the generation of a high volume of polyethylene wear microparticles (average diameter of 0.38 [53] to 1.33 μm [54]) that have been linked to elevated levels of osteolysis, also called the “particle” disease [55]. Osteolysis can be described as an uncontrolled inflammatory response around the implant that results in the resorption of the surrounding bone and eventual aseptic loosening of the device [56,57].

### 2.2 Second & third generation of total hip replacement implants

MoM implants were reintroduced to counteract the “particle disease”, after examination of unrevised devices retrieved during autopsies showed considerably lower wear volumes for these surfaces compared to MoP [58,59] (linear wear rate of ∼0.005 mm / year [60,61], Table 1). With improved manufacturing techniques, the second-generation MoM and ceramic-on-ceramic (CoC) implants offered two hard-on-hard bearing surface options that generated lower wear rates and good biomechanical properties [[62], [63], [64]].

Still, these bearing surfaces are not without risk. For example, CoC implants have a reported risk of fracture in the range of 0.004%−1.1% [[65], [66], [67]], and squeaking problems in the range of 1–31% [[68], [69], [70], [71]]. MoM implants result in the generation of wear nanoparticles and other metallic degradation products (i.e., reacted/corrosion metal ions, oxides, phosphates), with average size of < 80 nm, but probably lower due to difficulties measuring particles < 10 nm and dissolved ions [72,73], that have been associated with the potential for local tissue reactions [74] (Table 1).

A new generation of MoP surfaces was also developed to try to address the issue of high wear volumes, by changing the UHMWPE used for the acetabular cup for various types of cross-linked polyethylene [75,76]. Better wear resistance from cross-linked polyethylene helped reduce the wear volume (linear wear rate of 0.003 mm/yr [77] to 0.000 mm/yr [78]) and particle size (0.02 to 0.159 μm in diameter [[79], [80], [81]]) generated in the implantation site (Table 1). More recent analytical techniques used to isolate and identify polyethylene particles indicate that the submicro- and nano-particle volume generated by MoP implants is higher than previously thought [21,82].

**Table 1:** Summary of main characteristics of total hip replacement bearing systems.

**Bearing System**	**Wear Rates (Linear, mm/year)**	% Revision Rates at 10 Years*	**Particle Sizes**	**Ion Release Levels**	**Biological Responses & Other Comments**
Metal-on-Metal (MoM)	0.0054 (± 0.0019) [61]	17.76 (17.30–18.22) [83]	Mostly nanoparticles (∼<50 nm) [73]	Co (0.5 – 3.5 µg/L), and Cr (0.6 - 2.6 µg/L) in serum [84], and values of up to 4604 µg/g in dry tissue [85]	Nanoparticles and metal ions linked to local tissue reactions, inflammation, immune activation, ALVAL, ARMD. “Technical” ban in several countries due to lower performance compared to other bearings [86].
Ceramic-on-Ceramic (CoC)	0.0041 (± 0.0022) [61]	3.27 (3.17–3.37) [83]	5 nm to ∼300 nm (up to µm scale in some reports) [87]	Minimal ion release	Mechanical risks: fracture (0.004–1.1%) and squeaking (1–31%). Less inflammatory response and minimal in vivo detection. Higher cost [64,66,69].
New generations MoP (Cross-linked polyethylene)	0.0161 (± 0.0051) [61] 0.003 (±0.027) [77] **XLPE 2nd gen** = 0.068 (0 to 0.150) **XLPE 3rd gen** = 0.000 (0 to 0.091) [78]	**XLPE** = 1.24 (0.21 to 2.27) **HXLPE** = 1.47 (0.88 to 2.06) **CPE/UHMWPE** = 2.68 (2.13 to 3.22) *Note: 6.8-year revision rate* [88]	Smaller particles, 0.02 to 0.159 µm in diameter [[79], [80], [81]]	Negligible ion release	Reduced wear volume and particle size. Lower osteolysis risk, but still present [88].
First generations Metal-on-Polyethylene (MoP)	0.051 (±0.022) [77] **Low activity patients** = 0.093 (0.075 to 0.12) **High activity patients** = 0.103 (0.086 to 0.12) [50]	3.21 (3.12–3.31) [83]	Microparticles 0.38 to 1.33 µm [53,54]	Negligible ion release	Microparticles linked to osteolysis, macrophage activation, inflammatory response [57]

### 2.3 Revision rates of hip replacement implants

The technological advances in biomechanical and bearing surface design, as well as hybrid materials development have resulted in improved performance and success rates for most MoP, CoC and MoM implants [7,17,63]. Throughout the years, several multicenter analyses have been implemented for different products in different populations, corrected or not for age, showing that all bearing surfaces have relatively small and similar estimated rates of revision between 2 and 5% at 10 years, and a revision rate of around 10 to 15% at 15 or more years, with revision for aseptic loosening as the end point [10,17,83,[88], [89], [90]] (Table 1).

MoM implants seem to be the outlier, with revision rates that are comparable to the rest of the bearing systems in the first 3 years, regardless of the type of fixation (e.g., cemented, uncemented, hybrid), but quickly exceed the 5% mark at 10 years and even after 5 years in the case of uncemented fixation [83] (Table 1). The revision and study of these implants that are reaching the expected lifetime of >15 years, will provide a better understanding of why they fail, and what role do wear particles and metal degradation products from the bearing surfaces versus the trunnion play in this process, helping design better implants and improve success rates for patients.

## 3 Not all wear degradation products are created equal

The cytotoxicity of wear degradation products and their involvement in inflammation and aseptic loosening have always concerned orthopedic surgeons and implant manufacturers, as can be seen in some of the earliest published literature on the topic [40,91]. However, the wear degradation product characteristics that elicit a stronger immunological response, and lead to osteolysis and implant revision are still controversial.

**Fig. 2:**
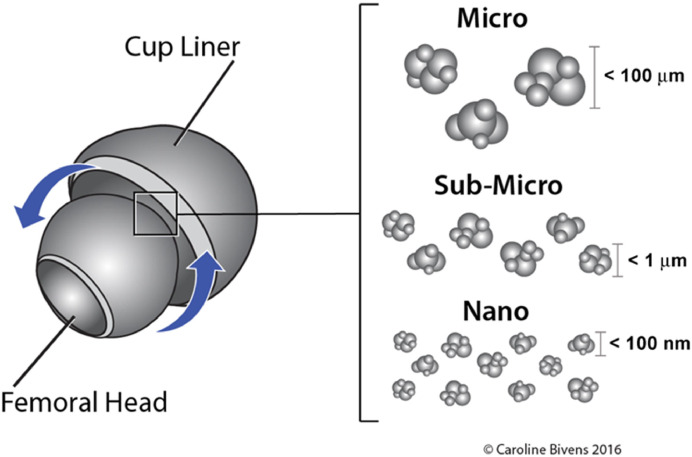
**Hip implant wear degradation products.** Schematic presenting the process of wear degradation product generation at different size scales: micro-, submicro- and nano-scale particles.

Across bearing couples, the size distribution of wear degradation products is large but typically nanoscale-skewed, with a mode that depends strongly on the source (Fig. 2). This means that nanosized particles dominate in numbers; while regarding mass or surface area, the fewer, larger particles contribute disproportionately. Metal-on-metal (MoM) bearings generate predominantly nanoparticles (often <50 nm) with a long tail into the submicron range [73]. Corrosion-dominated conditions and edge-loading can shift distributions toward larger agglomerates and plate-like fragments [92]. In contrast, metal-on-polyethylene (MoP) bearings shed mostly submicron UHMWPE particles (0.02 to 0.159 µm in diameter for highly crosslinked polyethylene [[79], [80], [81]]; 0.38 to 1.33 µm for 1st generation implants [53,54]). Ceramic-on-ceramic (CoC) bearings exhibit very low wear, typically submicron particles and scarce under standard conditions (5 nm to 300 nm [63,87]), consistent with excellent long-term performance. Importantly, modular head–neck (taper) junctions introduce an additional particle source with broad, multimodal size distributions—from nanometer-scale corrosion products to micron-scale fretting debris—that differ from MoM bearing-surface particles and can dominate local wear degradation products in trunnionosis scenarios [87,93,94].

Regarding the chemistry of wear degradation products, recent synchrotron work has sharpened the picture that most metal wear debris is not elemental metal but rather oxidized reaction products whose chemistry depends on where and how they formed. In a two-case tissue study using synchrotron X-ray fluorescence microscopy with XANES speciation (element-specific “fingerprints”), Liu et al. [95] mapped intracellular debris within macrophages and identified three dominant chromium-containing moieties in MoM tissues: Cr₂O₃, CrPO₄, and an alloy–oxide mixture—plus titanium in mixed crystalline/amorphous oxide states; they also quantified intracellular Co, Cr, Ti, and V at the femtogram scale per cell region, underscoring true in situ uptake and transformation (sXFM/XANES) rather than contamination artifacts. These findings extend earlier tissue-level speciation studies showing Cr(III) phosphate as the prevalent form around failed MoM implants (with cobalt more soluble/complexed), and Cr₂O₃ enrichment when fretting corrosion dominates at modular taper junctions [32]. Tribocorrosion can occur at both bearing and modular taper interfaces, although more studies comparing tribocorrosion wear from these surfaces are needed to understand if they leave chemically distinct fingerprints that likely drive different tissue responses (e.g., ALTR/ALVAL in soft tissues vs classical osteolysis with UHMWPE).

Complementary synchrotron analyses in peri‑implant bone and marrow further show log-normal, nanoscale-skewed particle distributions with evidence of cobalt release via de-alloying during particle aging; again pointing to speciation and ongoing transformation as key determinants of bioavailability and toxicity rather than “metal vs not” alone [96,97]. Together, this speciation-aware view supports the practical recommendation that both research models and clinical interpretation should (i) differentiate MoM bearing debris from taper-corrosion products, (ii) match test articles to in-tissue chemistries (CrPO₄/Cr₂O₃/Co complexes; Ti oxides), and (iii) report dose using ion levels, particle number/size/surface area, and flux (dose-per-time) so that links between chemistry, dose, and tissue phenotype can be compared across studies and bearing systems [95,98].

## 4 Biological response to wear nanoparticles and other degradation products

Before analyzing the cellular response, it is crucial to distinguish the histological finding of ALVAL from the broader clinical syndrome of ARMD. While ALVAL represents a specific T-cell mediated hypersensitivity response visible under the microscope, ARMD encompasses the full spectrum of trunnionosis-associated failure, including metallosis, pseudotumor formation, and direct chemical necrosis caused by high-flux ion release [36]. For the clinician managing a MoM patient, ARMD is the presenting phenotype—often mimicking infection—while ALVAL is the underlying pathological mechanism in a subset of these cases.

### 4.1 Hip periarticular microenvironment and cell targets

The tissue surrounding the hip joint is very heterogeneous, as it contains bone, blood vessels, lymphatic vessels, ligaments, nerves and other components. Similar to the concept of the knee as an organ [99], the hip joint and its associated tissues can be thought of as an organ when responding to a hip replacement implant. Thus, wear particles and other degradation products generated at the bearing surfaces of MoM or MoP implants will interact with a variety of cells, including macrophages, osteoclasts, osteoblasts, endothelial cells, fibroblasts and others [[100], [101], [102]]. Depending on size and chemistry, cells can internalize, traffic, or be functionally modulated by particles, with outcomes ranging from adaptive remodeling to apoptosis or necrosis [[102], [103], [104], [105], [106]].

### 4.2 Size- and route-dependent fate of wear degradation products

**Fig. 3:**
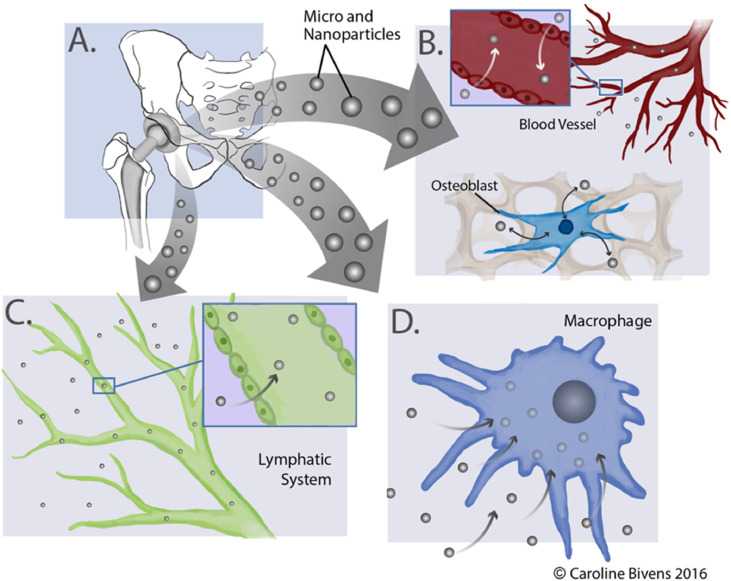
**Implant wear degradation product fate in the body.** (A) Micro- and nano-scale wear degradation products can be (B) actively phagocytosed by macrophages; (C) sequestered by the interstitial fluid and cleared by the lymphatic system; or taken up by the surrounding tissue (e.g., osteoblasts) and vasculature.

The size and the shape of the particles is a key parameter that determines the way they will be managed by the body (Fig. 3). Particles smaller than 10 nm are preferentially reabsorbed into the blood capillaries (Fig. 3B), while particles in the range of 10 to 100 nm favor lymphatic uptake (Fig. 3C) [107]. Larger particles, such as the 1 to 3 µm wear particles seen with UHMWPE bearing surfaces or metallic surfaces that have critically failed, also end up in the lymphatic system but with a slower uptake rate [[108], [109], [110]], probably due to slower traffic through the interstitial space via transport by macrophages (Fig. 3D).

As is the case for any bearing surface in a hip replacement implant, small traces and wear degradation products of metal / polyethylene / ceramic are found in the blood stream and organs distant from the implant site, such as the liver and kidneys [111,112], indicating that the excretory system is handling the waste generated by the implant.

### 4.3 Macrophage clearance and inflammatory activation

Macrophages are mostly known for a quick and sometimes aggressive response to the presence of wear degradation products as part of the normal inflammatory response [86,109]. However, macrophages are also capable of putting in place a strong anti-inflammatory mechanism to avert self-damage and they play a very important role in tissue homeostasis [113,114]. Macrophages are designed to phagocytose (i.e., ingest) large quantities of dead cells and other debris, including organic and metallic particles that need to be cleared or recycled [113,115]. This constitutive ‘clearance program’—illustrated by daily erythrocyte turnover and iron handling—operates with tight control of metal ions and minimal pro-inflammatory signaling [[115], [116], [117], [118], [119]]. A similar janitorial role is performed by macrophages with all the cellular debris generated after tissue remodeling from an injury or with hip implant wear degradation products [113,114,120,121].

In the case of MoP implants, several in vitro studies have examined the responses of macrophages to micro-sized UHMWPE particles [19,21,108]. In contrast, only in the last few years have we learned more about nanoparticles and other wear degradation products in hip replacement implants [20,122]. Results show a “critical size” range within the micro- and submicro-scale for particles that cause most macrophage activation, as opposed to nanoparticles or particles larger than 10 µm in diameter that are harder to phagocytose [21,123,124]. For example, Liu et al. [125] showed that nano-sized polyethylene wear debris did not activate peripheral blood mononuclear cells (PBMCs), but micron-sized polyethylene wear debris caused significantly elevated osteolytic cytokine release from PBMCs.

Similarly, larger metallic particles have usually been the ones implicated in higher macrophage activation related to MoM implants [126,127]. Indeed, many of the studies on inflammation caused by metallic wear particles performed in the 1990s showed considerable activation of macrophages after being exposed to microparticles [128,129]. More recent studies confirmed that larger microparticle size coupled to irregular shapes could lead to stronger inflammatory reactivity in human monocyte/macrophages [127].

Interestingly, the particle size most representative from MoM wear degradation products may be the more abundant nanoparticles generated during the regular low-volume wear of metal-on-metal bearing surfaces. More recently, metallic nanoparticles and metal ions have been found in the periprosthetic tissue, shown to interact with the phagosomes of living macrophages, elicit an immune response (pseudotumors) and lead to necrosis, clinically. However, in vitro studies evaluating the effect of metallic nanoparticles on macrophage activation or cellular cytotoxicity commonly find a response with doses that are much higher than what would be generated by a hip implant in the periprosthetic tissue [[130], [131], [132]].

### 4.4 Molecular mechanisms of cell-wear degradation products interactions

At the cellular level, nanoscale metal degradation products engage a multistep injury–danger program that integrates ion/particle uptake, intracellular corrosion, and ion-dependent signaling. Following phagocytosis by macrophages (and, to a lesser extent, by osteoblasts and fibroblast-like synoviocytes), particles traffic to acidified lysosomes, where local pH and chelators promote intracellular corrosion with release of Co²⁺ (highly soluble) and Cr predominantly as insoluble oxides/phosphates [98,133]. Lysosomal swelling and membrane permeabilization liberate cathepsins and mitochondrial danger signals, providing the proximal triggers for NLRP3 inflammasome assembly, caspase-1 activation, and pyroptotic IL-1β/IL-18 release [[134], [135], [136]].

In parallel, particle and Co²⁺ exposures amplify reactive oxygen species (ROS), collapse mitochondrial membrane potential, and activate NF-κB and MAPK (p38/JNK/ERK) cascades, up-regulating TNF-α, IL-6, and chemokines that recruit additional myeloid cells and osteoclast precursors [133,137]. At higher cobalt doses, lipid peroxidation and iron-handling dysregulation converge on ferroptosis-like programs, while DNA damage and ATR/Chk signaling (i.e., a DNA-damage checkpoint pathway that cells use to sense trouble during DNA replication and pause the cell cycle so damage can be repaired) contribute to growth arrest and cytotoxicity in non-myeloid cells [137,138].

The immunologic context is further shaped by macrophage polarization dynamics, with M1-skewed profiles (i.e., pro-inflammatory) predominating near high particle/ion burdens and M2-repair programs (i.e., anti-inflammatory) emerging after debridement or with lower, intermittent fluxes [136]. Importantly, protein–metal adducts can act as neoantigens in genetically susceptible hosts (e.g., specific HLA-DQ haplotypes), supporting T-cell–mediated delayed-type hypersensitivity and the ALVAL phenotype, which is consistent with the soft-tissue necrosis and lymphocytic infiltrates more characteristic of metal degradation products than classic UHMWPE-driven osteolysis [139]. Together, these linked pathways that involve lysosomal destabilization, NLRP3/pyroptosis, ROS–NF-κB/MAPK–driven cytokine programs, dose-dependent ferroptotic/DNA-damage signals, and adaptive immunity in HLA-primed patients, provide a coherent mechanistic framework that connects speciation and dose-rate to the histopathology and clinical course of adverse local tissue reactions [98,134,139].

Finally, one additional pathway that may be involved is that, under conditions of low oxygen, tissue homeostasis is promoted by macrophages through different mechanisms such as the activation of the hypoxia-inducible factor (HIF) pathway [[140], [141], [142]]. HIF-inducible genes have been associated with important homeostatic processes, such as angiogenesis [[143], [144], [145]] and cellular metabolism [141,146]. Interestingly, cobalt ions and nanoparticles can mimic hypoxia conditions that stabilize HIF-1α even in the presence of oxygen and lead to a sustained inflammatory response [140,147,148]. Accordingly, a study found higher protein expression of HIF-1α in peri‑implant human tissue samples from patients that underwent primary revision surgery for MoM implants compared to MoP devices, with the authors suggesting this could be one mechanism to explain the unusual reactions around some implants, such as excessive fibrous tissue growths or “pseudotumors” [149,150].

## 5 In *vitro* models of hip implant failure: difficulties simulating *in vivo* conditions

Several studies have looked at the effects of metallic wear degradation products (e.g., particles, corrosion ions, reacted oxides, phosphates) on macrophages, suggesting higher activation and cytotoxicity with increasing concentration [109,[151], [152], [153]]. However, these studies typically do not represent the size, chemistry and nature of the wear degradation products generated by MoM bearing surfaces, nor do they represent concentrations comparable to what macrophages in the joint experience in cellular timescales, which go from seconds to hours [154]. For example, hip aspirates from MoM arthroplasties have shown total amount of metal (i.e., cobalt and chromium) anywhere from 1.4 to 4604 µg/g in periprosthetic tissues [85], respectively, but several studies have seen macrophage activation only when they reach doses that are 10 to 200 times [152] or even 2500 times larger [151].

Some hypotheses suggest that excessive particle phagocytosis may disrupt the phagolysosomal membrane of macrophages and overwhelm the inflammatory immune response [155,156]. Although high estimates calculate trillions of nanoparticles generated over a year’s time [157,158], cellular processes need to be evaluated in seconds to minutes. If one assumes a load of one trillion particles over a year, on a per-second basis the load would be 32,100 particles. Uptake by one macrophage can reach up to 5000 nanoparticles [159,160]. Thus, performing a very simplistic approximation on a volume-basis alone, <10 macrophages on a per-second basis, and a few hundred of the millions available on a per-minute basis, should be competent to manage the number of particles that can be present around the hip joint due to normal wear.

It is also important to consider that nanoparticles and other wear degradation products may be taken up by cells other than macrophages, which could help clear them from the hip joint more quickly. Studies have shown that osteoblasts can also endocytose some of these particles [161]. In the same study, similar endocytosis results were found for UHMWPE, commercially pure titanium, and titanium-aluminum-vanadium alloy, suggesting that this is a well-conserved response that does not depend on the chemistry of the bearing surface wear particles. In other words, this phenomenon would occur regardless of the bearing material.

The chemistry of the wear particles and other degradation products is another parameter that can affect the response of macrophages, as some ionic species could be more cytotoxic than others [73]. However, to compare in vitro simulations with in vivo or clinical scenarios in a reliable scientific analysis and draw definitive conclusions, two criteria should be met: (1) appropriate and comparable doses need to be used to assess the cytotoxicity; and (2) the cells must be cultured under conditions where the extracellular fluid is buffered and not static, just as it is in the body.

## 6 Dose and rate are key to understanding cell viability and implant survivorship

The concepts of dose (particle/ion concentration per unit volume) and rate (number of particles/ions per unit volume per unit time) need to be clarified to understand the effect of nanoparticles on biological response. Dose can also be considered as the total concentration of an ionic species/number of particles that the tissue has accumulated over some time. The dose rate is the concentration/number of particles/ions being emitted at a given period (e.g., seconds, minutes, hours), and it is a key parameter for cellular activity. These definitions make evident the inherent difficulties encountered when trying to model tissue reaction to wear degradation products, because the dose may be measured from biopsies of the joint but the dose rate around the cells of interest can only be calculated rather than directly measured. Implant wear degradation products are hardly constant, with higher wear rates within the first year of the implant’s life [60,162], making the prediction of a dose rate very difficult. In vitro studies are commonly performed using the measured doses (or higher) [151,152], which can shed light on important aspects of maximum concentration tolerance, but risks could be misinterpreted.

For example, in a study [152] that examined the inflammatory effect of nanoparticles, the particles used were commercially available cobalt (99.8%) and chromium (99%) particles, which are not present in MoM patients. The cobalt containing particles in MoM patients are either small flakes from the oxide layer containing mainly chromium oxides (i.e., no presence of cobalt) or larger particles that mirror the chemical composition of the bulk material [32,163,164], almost in a 2:1 ratio (approximately 65% Co, 28% Cr, and 5% Mo). When properly calculated and normalized to µg of cobalt per g of cells, Kwon et al. [152] actually used 1022,492 µg/g. This number is >220 times higher than the highest concentration at the tissue level that was observed by Lohmann et al. [85] in MoM patients (4604 µg/g). For these reasons, in vitro studies and general assumptions should be considered carefully when drawing conclusions regarding potential responses in MoM patients.

To make in-vitro studies more relevant to what actually happens around implants, experiments should replace one-time, high-dose additions in static wells with exposures that match both the chemistry and the pace of release in vivo: that is, using degradation products with the right speciation (i.e., the actual chemical forms found in tissues) delivered at a realistic flux (dose-per-unit-time) in protein-containing media that allow a natural protein corona to form on particles (i.e., a coating that changes how cells see and internalize them). In order for results to be compared across platforms, doses should be reported with complementary metrics: ion concentration, particle number and size distribution (e.g., D10/D50/D90), surface area, and cumulative dose rate over time [32,98].

Perfused microphysiological systems and organ-on-chip co-cultures (miniaturized devices with continuous flow that impose physiologic shear and oxygen/nutrient gradients) help prevent artifactual accumulation seen in static plates and better preserve cell-to-cell crosstalk among macrophages, fibroblasts, and osteoblasts; they also let us model time-staged exposures such as chronic low-level tribocorrosion versus intermittent taper fretting [[165], [166], [167]]. Biomarker measurements should go beyond bulk cytokines by adding single-cell RNA-seq/CyTOF (to resolve responsive subpopulations) and spatial transcriptomics/proteomics (to map pathways like HIF-1α, NF-κB, and NLRP3 to specific micro-niches in 3D cultures), which has already clarified myeloid–fibroblast heterogeneity in human joint tissues [[168], [169], [170], [171]]. Finally, materials verification should confirm that wear degradation products to be evaluated truly mirror in-tissue chemistry; for example, with synchrotron spectroscopy (XANES/EXAFS; element-specific “fingerprints”) or ToF-SIMS (mass-spectrometry imaging of surfaces), so mechanistic links between chemistry, dose/flux, and tissue outcomes rest on realistic inputs [95,98].

## 7 Individual susceptibilities

Another aspect that is difficult to consider when predicting clinical outcomes from in vitro or in *vivo* results is the concept of individual susceptibility and human genetic variations. This biological variability has been well established in the literature and has been linked to genetic differences between patients, resulting in a range of responses, from susceptibility to diseases such as tuberculosis [111] to pharmaceutical adverse reactions [172,173]. Interestingly, a study evaluating human individual susceptibility to fine particulate air pollutants found that lifestyle factors such as fruit and vegetable intake, meat consumption and smoking had an even greater weight on the inflammatory response of lymphocytes than genetic factors [174]. Thus, individual susceptibility, either from genetic or behavioral factors, could translate to a patient’s general joint health and its reaction to implant wear degradation products.

The musculoskeletal field is only recently beginning to understand that sex differences in disease prevalence or severity are a result of biological differences at the cellular and molecular level that go beyond hormonal levels [175,176]. Furthermore, not only can the prevalence of disease be different between sexes but the response to therapy also can be just as diverse. A good example relates to the evident sex differences in osteoarthritis, with post-menopausal women suffering greater severity of osteoarthritis in most joints than men of the same age [[177], [178], [179]]. In addition, a few studies have established that females exhibit increased levels of implant failure possibly due to adverse local tissue reactions, implant dislocation, aseptic loosening, and metal hypersensitivity responses [[180], [181], [182]].

Hypersensitivity to metallic wear ions and particles from implants, which involves a delayed inflammatory reaction that results in lymphocyte activation, is another hip replacement complication reported in the literature [[26], [27], [28],^110^]. Metal hypersensitivity reactions have been found in 10–15% of the general population, ∼20–30% in well-functioning implants and higher (40–60%) in poorly functioning/failed implants, although a causal effect between hypersensitivity and implant failure is yet to be established and failure directly attributable to hypersensitivity remains rare (<1%) [[183], [184], [185], [186]]. Importantly, although some studies have found evidence to suggest MoM implants cause reactivity through an elevated ion dose-type induction found mainly under conditions of abnormal wear [152,187], these results do not rule out dose-dependent innate and adaptive immune responses that may be dependent on patient individual susceptibilities (i.e., patient A may have metal reactivity at a certain ion level whereas patient B does not) [27,110,187]. The delayed nature of this response, coupled to the relatively small number of patients that suffer from it, agrees with the lack of evidence to indicate that nanoparticles are in part responsible for hypersensitivity that will lead to inflammation resulting in revision surgeries.

Several theories have been put forward to explain this phenomenon, including apoptosis of macrophages induced by Co and Cr ions [[188], [189], [190]]. This is hypothesized to lead to recruitment of new macrophages to manage the site, and ultimately to a foreign body macrophage response [191]. Additional hypotheses include ALVAL/type IV hypersensitivity reactions and the concept of degradation products serving as haptens to stimulate immune responses. The number of studies addressing these issues has increased over the past few years, and the results will eventually provide a more comprehensive understanding of how particles of many kinds elicit biological responses in different patients.

**Fig. 4:**
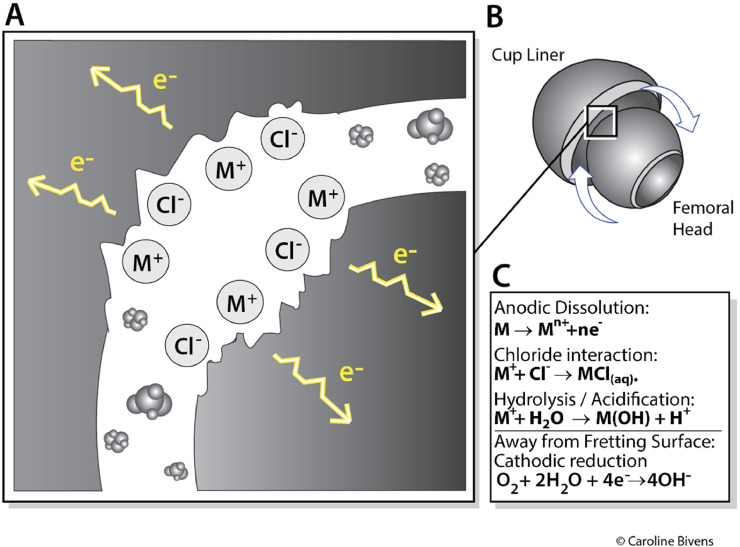
**Mechanistic overview of tribocorrosion in bearing surfaces and modular junctions.** (A) Schematic of the electrochemical environment within a crevice (i.e., (B) the geometry of the bearing surfaces, or the head-neck taper where these forces occur). Mechanical fretting disrupts the passive film, promoting localized anodic dissolution (M → Mⁿ⁺ + ne⁻) and ingress of chloride ions (Cl⁻) to maintain charge neutrality. (C) Hydrolysis of released metal ions generates protons (H⁺), leading to acidification and enrichment of metal species within the crevice, which can accelerate corrosion and influence local tissue exposure (e.g., inflammation, ARMD). Because oxygen is depleted within the crevice, cathodic oxygen reduction occurs on nearby, better-oxygenated regions of the implant surface outside the crevice (differential aeration). The electrochemical circuit is completed by electron transport through the metal and ionic conduction through the electrolyte, producing localized interfacial current densities that shape pH and speciation.

Other less-discussed parameters, such as localized electrochemical current densities (charge-transfer rates) associated with tribocorrosion at the implant–electrolyte interface, could also influence peri‑implant biology and play a role in implant failure, as studies have shown that electrical signals (i.e., currents, electric potentials) can also have a detrimental effect on cell viability in vitro (Fig. 4) [192,193]. Electrochemical corrosion involves local interfacial currents (current densities) associated with anodic metal dissolution and cathodic reduction reactions on the implant surface [194]. In crevice geometries, a differential aeration cell can form [195,196]: oxygen is depleted inside the crevice, promoting anodic dissolution there, while cathodic oxygen reduction occurs on adjacent, better-oxygenated metal surfaces. The circuit is completed by electronic current within the metal and ionic current in the surrounding fluid, leading to local pH and chemistry shifts (e.g., acidification via hydrolysis) that influence speciation and tissue exposure. Importantly, these are localized electrochemical processes at the implant–fluid interface and should not be interpreted as macroscopic ‘electrical currents circulating’ through the body.

Chemical differences between the materials in the load-bearing surface and their insulating nature (i.e., metal is an electric conductor, while polyethylene and ceramic are not) could add to the complexity of the electrochemical reactions in the joint space and the biological response in each patient [192,197,198]. Still, a long road of studies waits ahead to confirm these hypotheses in vivo and clinically, and to elucidate their mechanisms.

## 8 Future outlook for hip replacement implants

### 8.1 Conclusions

Evidence from retrievals, tissues, and simulators confirms that all hip implants release a chemically diverse mix of degradation products. These range from chromium(III) oxides, phosphates, and soluble cobalt species typically associated with metal alloys, to UHMWPE and occasional ceramic particulates. Crucially, these products differ in speciation, size distribution, and dose rate, thereby eliciting distinct biological responses: soft-tissue–predominant reactions, which are clinically manifested as Adverse Reactions to Metal Debris (ARMD), are typical of metal degradation products; whereas macrophage-driven osteolysis is classically linked to polymeric wear. Much of the inconsistency across historical studies stems from experimental models that failed to match the specific in-tissue chemistry or exposure kinetics of late-stage failures. Converging on speciation-matched inputs and physiologically realistic dosing is therefore essential to reconcile these discrepancies and sharpen the mechanistic link between material release, cell behavior, and clinical outcomes.

### 8.2 Future perspectives & research directions

To better predict the longevity of current and older devices, future inquiries must mirror the actual chemical forms found around implants (i.e., the corrosion products of trunnionosis) rather than relying on idealized surrogates. Research reporting should adopt complementary metrics that enable inter-laboratory comparison, including ion concentration, particle number, surface area, and dose rate (flux). Furthermore, advancements in surface analysis must be leveraged to characterize the protein corona and agglomeration states that dictate bioavailability. To move beyond static, high-bolus exposures that obscure time-dependent toxicity, the field must adopt perfusion bioreactors and microfluidic organ-on-chip co-cultures. These platforms impose physiologic shear and nutrient gradients, allowing for time-staged exposures that mimic chronic low-level tribocorrosion versus the intermittent high-flux release of taper fretting. Such dynamic models better preserve the crosstalk among macrophages, fibroblasts, osteoblasts, and endothelial cells, reducing artifactual accumulation and providing a more accurate window into the peri‑implant microenvironment.

This methodological evolution should be paired with high-resolution analytics. The improved accessibility of single-cell RNA-seq and CyTOF now allows researchers to map functional readouts, such as macrophage polarization and osteoblast differentiation, to specific micro-anatomic niches in 3D constructs. Linking these cell states to verified materials chemistry will clarify which combinations of speciation and flux drive the transition from homeostasis to the necrotic and inflammatory pathways characteristic of ARMD. Simultaneously, lessons learned from metal-on-metal systems should continue to guide materials engineering, particularly in optimizing alloy microstructure and modular junction designs to minimize the electrochemical susceptibility of head-neck tapers.

Finally, these mechanistic insights must translate into systemic changes in how we monitor patient populations globally. The challenge of “traveling retired” patients demands a revolution in longitudinal surveillance to close the loop on the "lost-to-follow-up" paradox. Current National Joint Registries track revision rates based on local data, creating a statistical blind spot when patients migrate. For instance, when a retiree moves to Panama or Costa Rica and undergoes revision surgery for late-stage ARMD, the original registry often records that patient as "lost to follow-up" or erroneously as a successful survivor. Future efforts require cross-border data sharing protocols and the development of "implant passports" that allow surgeons in receiving countries to identify the specific metallurgy of an older device to anticipate ARMD risks before opening the patient. By integrating lab-grade dose metrics with registry-linked prospective cohorts, the field can better connect biological mechanisms to patient outcomes, ensuring that diagnosis and treatment remain effective even as patients move across borders.

## Funding

This work was supported by SENACYT, Panama [grants # ITE15–016, PFID-INF-2020–43, and SNI-SENACYT contracts SNI-12–2020 and SNI-051–2023 for RAG; contract DDCCT-No- 270–2020–122 for VRL] and personal funds (VRL). The work was also supported by the National Institutes of Health NIAMS-USPHS [Award Nos. AR052102 and AR068703 for BDB and ZS]. Sponsors were not involved in study design.

## Disclosures

RAG and BDB have received remuneration for their expert testimony in a legal case involving a hip replacement implant company.

## Declaration of generative AI and AI-assisted technologies in the writing process

During the preparation of this work the authors used a private ChatGPT account in order to double-check some of the calculations in the manuscript and check for the most updated and relevant references in some sections. The tool was not used to generate content in the manuscript. After using this tool, the authors reviewed and edited the content as needed and take full responsibility for the content of the published article.

## CRediT authorship contribution statement

**Rolando A. Gittens:** Writing – review & editing, Writing – original draft, Visualization, Methodology, Investigation, Formal analysis, Data curation, Conceptualization. **Venettia R. Leslie:** Writing – review & editing, Visualization, Investigation, Formal analysis. **David J. Cohen:** Writing – review & editing, Visualization, Validation. **Zvi Schwartz:** Writing – review & editing, Validation, Supervision, Resources, Methodology. **Barbara D. Boyan:** Writing – review & editing, Writing – original draft, Supervision, Resources, Methodology, Conceptualization.

## Declaration of competing interest

The authors declare the following financial interests/personal relationships which may be considered as potential competing interests: Barbara D. Boyan & Rolando A. Gittens reports financial support was provided by Barnes & Thornburg LLP. If there are other authors, they declare that they have no known competing financial interests or personal relationships that could have appeared to influence the work reported in this paper.

## Data Availability

No data was used for the research described in the article.
